# NMR-based metabolic profiling in healthy individuals overfed different types of fat: links to changes in liver fat accumulation and lean tissue mass

**DOI:** 10.1038/nutd.2015.31

**Published:** 2015-10-19

**Authors:** A Elmsjö, F Rosqvist, M K R Engskog, J Haglöf, J Kullberg, D Iggman, L Johansson, H Ahlström, T Arvidsson, U Risérus, C Pettersson

**Affiliations:** 1 Department Medicinal Chemistry; Analytical Pharmaceutical Chemistry, Uppsala University, Uppsala, Sweden; 2 Department of Public Health and Caring Sciences; Clinical Nutrition and Metabolism, Uppsala University, Uppsala, Sweden; 3 Department of Radiology, Oncology and Radiation Sciences; Radiology, Uppsala University, Uppsala, Sweden; 4 Medical Product Agency, Uppsala, Sweden

## Abstract

**Background:**

Overeating different dietary fatty acids influence the amount of liver fat stored during weight gain, however, the mechanisms responsible are unclear. We aimed to identify non-lipid metabolites that may differentiate between saturated (SFA) and polyunsaturated fatty acid (PUFA) overfeeding using a non-targeted metabolomic approach. We also investigated the possible relationships between plasma metabolites and body fat accumulation.

**Methods:**

In a randomized study (LIPOGAIN study), *n*=39 healthy individuals were overfed with muffins containing SFA or PUFA. Plasma samples were precipitated with cold acetonitrile and analyzed by nuclear magnetic resonance (NMR) spectroscopy. Pattern recognition techniques were used to overview the data, identify variables contributing to group classification and to correlate metabolites with fat accumulation.

**Results:**

We previously reported that SFA causes a greater accumulation of liver fat, visceral fat and total body fat, whereas lean tissue levels increases less compared with PUFA, despite comparable weight gain. In this study, lactate and acetate were identified as important contributors to group classification between SFA and PUFA (*P*<0.05). Furthermore, the fat depots (total body fat, visceral adipose tissue and liver fat) and lean tissue correlated (*P*(corr)>0.5) all with two or more metabolites (for example, branched amino acids, alanine, acetate and lactate). The metabolite composition differed in a manner that may indicate higher insulin sensitivity after a diet with PUFA compared with SFA, but this needs to be confirmed in future studies.

**Conclusion:**

A non-lipid metabolic profiling approach only identified a few metabolites that differentiated between SFA and PUFA overfeeding. Whether these metabolite changes are involved in depot-specific fat storage and increased lean tissue mass during overeating needs further investigation.

## Introduction

Obesity, especially liver and visceral fat accumulation, has been proposed as a causative factor in the development of multiple metabolic disorders, for example, type 2 diabetes.^1^ According to recent cross-sectional^2, 3, 4^ and interventional data,^5^ it has been suggested that dietary fat composition could have a key role in ectopic fat deposition, that is, saturated fatty acids (SFA) could promote hepatic steatosis, whereas polyunsaturated fat (PUFA) may be preventive. Recently, the randomized LIPOGAIN study showed that overfeeding diets with PUFA rather than SFA caused considerably less accumulation of fat in the liver, visceral region and total body, despite similar weight gain between the diets.^6^ Differential effects were observed in adipose tissue gene expression and fatty acid metabolism, but the mechanisms were still not clear, despite the fact that there was a significant association between the plasma and tissue fatty acids fed in the different diets. However, it was perceived that there might be an influence on pathways, beyond fatty acid metabolism, that could be of interest. In this regard, the influence of fatty acid type on non-lipid metabolites such as amino acids or intermediate metabolites in energy metabolism and substrate partitioning would be of great interest to explore.^7, 8, 9, 10^ Metabolic profiling, through the use of pattern-recognition techniques (that is, metabolomics), could potentially identify such relevant metabolites that would not have been detected otherwise.

Nutrition-based metabolomics studies are complicated owing to the inter- and intra-variability in metabolic pathways and digestion.^11^ It can therefore be challenging to identify correlations between consumed fat and changes in body composition when studying moderate differences in diet.

An efficient and simple way to extract non-lipid metabolites from plasma is to precipitate with cold acetonitrile, a procedure well established within bioanalytical chemistry. These metabolites can then be detected with nuclear magnetic resonance (NMR) and liquid chromatography hyphenated with mass spectrometry.^12^ Owing to the unique character of ^1^H NMR spectroscopy, its high reliability and the simultaneous detection of a broad range of metabolites, it is a particularly suitable technique for metabolomic studies.^13^

In the research presented here, a non-targeted NMR-based metabolic profiling approach was used for the identification and relative quantification of low-molecular weight non-lipid metabolites in plasma samples from the LIPOGAIN study. The primary aim was to study the possible differences in low-molecular weight non-lipid metabolites arising as a consequence of a diet high in SFA compared with high PUFA diet. The secondary aim was to correlate metabolic profile NMR data with observed magnetic resonance imaging (MRI) data of fat and lean tissue composition.

## Materials and methods

### Chemicals

All buffers were prepared with heavy water (D_2_O, 99.8%) bought from Amar Chemicals (Döttingen, Switzerland). The buffer components, K_2_HPO_4_ (99%) and KH_2_PO_4_ (99.5%), were both from Merck (Darmstadt, Germany), and 2,2-dimethyl-2-silapentane-5-sulfonate sodium salt (99.9%) and acetonitrile (99.9%) were purchased from Sigma Aldrich (St Louis, MO, USA).

### The design of the LIPOGAIN study

The protocol used in the LIPOGAIN study has been described in detail elsewhere.^6^ In brief, in a randomized double-blind trial, 39 human subjects (mean±s.d. age: 27±4, body mass index: 20.7±2.1) were overfed for 7 weeks with muffins high in SFA (palm oil) or n-6 PUFA (sunflower oil). Muffins were added to the habitual diet and their quantity was adjusted for the individuals involved to achieve a 3% weight gain. The individuals were instructed to keep their other dietary and lifestyle habits unchanged. Blood sampling was performed after an overnight fast (12 h), before and after 7 weeks of overfeeding. From each individual, 6 ml blood was drawn into a vacutainer tube containing K_2_-EDTA (Vacuette K_2_-EDTA, Mediq Sweden, Kungsbacka, Sweden). The samples were centrifuged for 10 min at room temperature before the plasma was dispensed into eppendorf tubes. After this, all samples were stored at −70 °C until analysis. All 39 individuals completed the intervention. Both groups consumed, on average, 3.1±0.5 muffins per day and gained equally in weight (1.6±0.85 vs 1.6±0.96 kg). The participants' physical activity level (measured using accelerometers) did not change or differ between the groups during the intervention. For each individual, the liver fat, visceral fat, total fat and lean tissue content were measured by MRI at the baseline and after 7 weeks.^6^

### Ethics

The study was conducted in accordance with the Declaration of Helsinki. All subjects gave written informed consent prior to inclusion, and the study was approved by the Regional Ethical Review Board of Uppsala, Sweden.

### Sample preparation

All blood plasma samples were prepared in a blinded and randomized order. Plasma was thawed at room temperature and homogenized by vortexing. Aliquots of 200 μl plasma were mixed with 600 μl of cold acetonitrile. The samples were then vortexed for 30 s and left at room temperature for 10 min; this was followed by centrifugation for 10 min (6720 *g*), whereupon 650 μl of the supernatant was evaporated at 36 °C under a stream of nitrogen. The samples were then reconstituted in 600 μl of 154 mM phosphate-buffered D_2_O (pD 6.8) containing 34 μM 2, 2-dimethyl-2-silapentane-5-sulfonate sodium salt (as a chemical shift reference), and stored at −80 °C until NMR analysis was performed.

### NMR analysis

NMR measurements were carried out at 298°K on a Bruker Avance 600 MHz (Bruker BioSpin GmbH, Rheinstetten, Germany) equipped with a cryoprobe. For each sample, the 1D NOESYPR1D standard pulse sequence (–RD-90°-t_1_-90°-t_m_-90°-ACQ) was used. Each pulse had a 90° pulse length; the total number of free induction decays recorded was 256, and these were collected into 32 K data points and zero filled to 64 K data points. The spectral width was set to 7183.91 Hz, giving an acquisition time of 4.56 s. The delay (t_1_) and the mixing time (t_m_) were set to 6 μs and 180 ms, respectively, and the relaxation delay (RD) was 3 s, resulting in a total acquisition time of 33 min for each sample.

### Data pre-processing

Each spectrum was multiplied by an exponential weighting function (0.15 Hz), Fourier transformed, phased, aligned to the 2,2-dimethyl-2-silapentane-5-sulfonate sodium salt signal, baseline corrected and divided into narrow bins (0.01 p.p.m.) using the ACD/Labs NMR tool (Version 12.0). Bins containing signals from water, 2,2-dimethyl-2-silapentane-5-sulfonate sodium salt and EDTA were excluded before each of the remaining bins was normalized to the total intensity. Two datasets were created, one used for studying the metabolic change comprised of the difference in each bin over the 7-week-long period, that is, the post-treatment bins had the intensity of the baseline bins subtracted from them for each individual. The second dataset was used for testing the data quality and the experimental setup and contained both the baseline and the post treatment bins.

### Statistical analysis

The datasets were pareto scaled before pattern recognition techniques were applied using SIMCA 13.0 (Umetrics, Umeå, Sweden). Principal component analysis (PCA) was used to give an overview of the data, enabling outliers to be identified, as well as trends and groupings; a second purpose was to control the quality of the design and the experimental setup. Orthogonal partial least square discriminant analysis (OPLS-DA) was used to identify the variables contributing to group classification. Interpretation of the OPLS models was carried out by using s-plots together with VIP plots (variable importance for the projection plots).^14^

The spectral peaks from metabolites identified as potential contributors to group classification were manually integrated and normalized to each spectrum's total intensity. For each metabolite, the post-intervention samples had the respective baseline samples subtracted from them. Students' *t*-tests (Microsoft Office Excel 2007) were used to determine whether there were any statistical differences between the two diets. Branched amino acids (BCAA) and the aromatic amino acids (AAA) were integrated as two integrals spanning δ 0.90–1.05 and 6.75–7.85 p.p.m., respectively.

### Metabolite identification

Assignments of NMR peaks were performed according to the Metabolomics Standards Initiative^15^ by comparing with literature,^16^ the Human Metabolome Database (V 2.5)^17^ and using 2D TOCSY experiments.^18^ Uncertain identities were confirmed by spiking plasma with reference substance and were performed for metabolites that were either contributing to a group classification or correlated with clinical outcomes. For all metabolites identified, more than one signal was used for identification with the exception of those metabolites that only display as singlets. The metabolites identified are putatively annotated compounds as according to the Metabolomics Standards Initiative nomenclature.^15^

### Correlation of body fat accumulation and metabolic profiling data

As previously described, MRI assessments were conducted before and after the intervention.^6^ The following assessments were significantly (*P<*0.05) different for the two groups: total body fat, liver fat, visceral adipose tissue and lean tissue, while a similar weight gain was observed.

Each of the above-mentioned MRI assessments was correlated with the normalized metabolites using OPLS models. The MRI assessments were used as the *y*-variable and the NMR-data as the *x*-variables. Interpretation and comparison between the models were made using shared and unique structure plots (SUS-plots), where the correlation of the scores from two separate OPLS models are compared.^14^ If two OPLS models show similar relationships between the *x*-variables and the single *y*-variable, the *x*-variables will form a straight line from the lower left corner to the upper right one, indicating shared structures, while *x*-variables along the *y* axis or the *x* axis will be seen as unique structures. Metabolites with a correlation coefficient (*P*(corr)) higher than 0.5 and a variable influence on projection (VIP) higher than 2.5 were considered to be correlating metabolites.^14^ Correlating metabolites were manually integrated and normalized to each spectrum's total intensity. The normalized integral from each metabolite were subjected to linear regression analysis (Microsoft Office Excel 2007) together with the MRI assessments.

## Results

**Figure 1:**
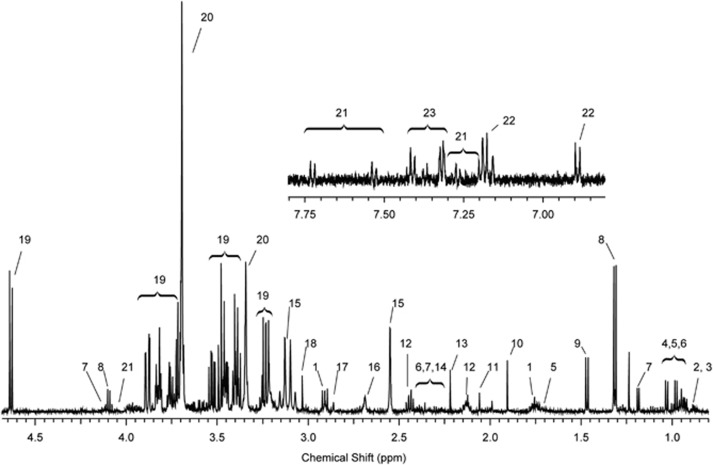
Typical NMR spectrum from a subject in the PUFA group. Good selectivity can be observed for a number of metabolites (for example, BCAAs,^4, 5, 6^ 3-hydroxybutyrate^7^ and lactate^8^) owing to the reduced protein and lipid signals.^27, 28, 29, 30^ For complete assignment see Table S1.

The metabolic profiling presented in this work aims to examine the differences between the SFA and PUFA group, the differences in metabolic profile within each group (week 0 and 7) as well as the correlation of metabolites to MRI data. A one-dimensional ^1^H-NMR spectrum and its annotation from an individual in the PUFA group is presented in Figure 1 and Supplementary Table S1. Visual inspection of all NMR spectra revealed no obvious class-specific differences in the metabolic composition, however, spectra from one test subject (both baseline and post treatment) showed some abnormal peaks that could not be explained by the clinical information. These spectra, together with one post-treatment spectrum with broad and intense lipid signals were excluded, resulting in 70 remaining spectra from 35 individuals.

To minimize the risk of confounding, PCA was used to identify the patterns related to age, baseline body mass index, gender and sample preparation date or acquisition date. No such patterns were observed, indicating that the data and the experimental setup were of adequate quality. The PCA model also demonstrated a gathered cluster for all the spectra with no outliers indicating correlating spectra.

**Figure 2:**
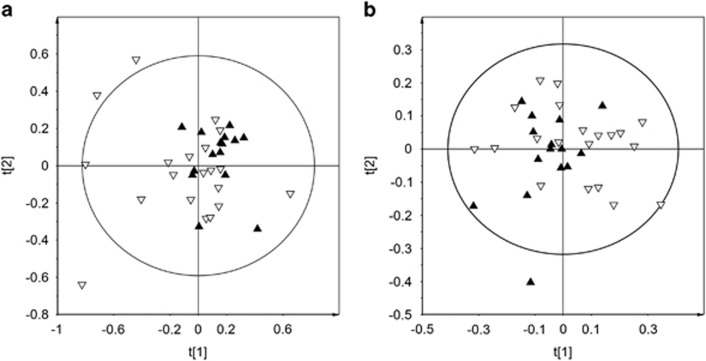
PCA plots of NMR data. (**a**) PCA model of all bins normalized to total NMR intensity with empty triangles representing SFA diet and inverted filled triangles representing PUFA diet (R^2^X=0.704, Q^2^X=0.450). (**b**) PCA model with glucose and lactate bins removed followed by data re-normalization where empty triangles represent SFA diet and inverted filled triangles represent PUFA diet (R^2^X=0.612, Q^2^X=0.225).

As expected, high inter-individual variation of glucose and lactate could be observed, and exclusion of glucose and lactate was carried out to identify other variables important for group (diet) discrimination. As expected, this lead to a decrease in model prediction power as manifested by the lowered Q^2^ value (Figure 2).

### Inter-group differences

OPLS-DA was used to locate metabolites that were important for group classification between the two diets. Examination of the corresponding s-line plot identified the metabolites contributing to group classification as 3-hydroxybutyrate, creatine/creatinine, acetoacetate, acetate, alanine, the AAAs (tryptophan, tyrosine and phenylalanine) and the BCAAs (valine, isoleucine and leucine).

Owing to a large inter-individual variability in the data, which could be expected in a moderate diet intervention, low R^2^- and Q^2^-values of the OPLS-DA model was observed. Therefore, to further confirm the differences between the two dietary groups, the NMR signal of each relevant metabolite, including lactate, was manually integrated, normalized to the total intensity and subjected to student's *t*-tests. The results of the *t*-tests indicated that lactate and acetate were significantly different (*P*<0.05) and also that 3-hydroxybutyrate differed between the PUFA and SFA diet (*P*-value=0.097). In summary, PUFA was associated with higher levels of lactate and lower levels of acetate and 3-hydroxybutyrate compared with SFA.

### Intra-group changes

**Figure 3:**
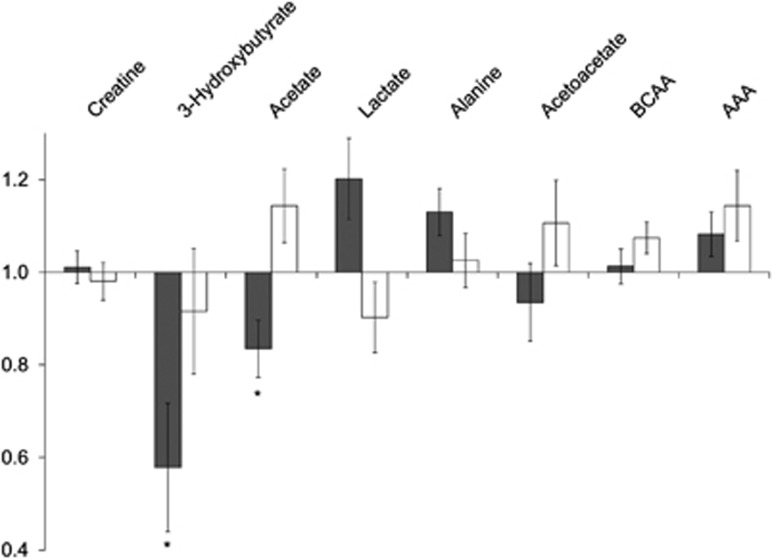
Relative change in key metabolites. Metabolites identified as contributing to discrimination between the PUFA (gray bars) and SFA diets (white bars) were manually integrated and subjected to students' *t*-tests. The bars represent the relative change in metabolite concentration over the study period, that is, a bar below 1 indicates a concentration decrease, while a bar above 1 indicate an increased metabolite concentration. The error bars represent s.e.m., while * indicates a significant change (*P*<0.05).

The metabolite changes within each dietary group are illustrated in Figure 3. For the PUFA group, the following metabolites tended to increase: lactate (*P*=0.088), alanine (*P*=0.075) and the AAAs (tryptophan, tyrosine and phenylalanine; *P*=0.095); while acetate and 3-hydroxybutyrate (*P*<0.05 for both) decreased significantly. For SFA, the BCAAs (valine, leucine and isoleucine; *P*=0.059) tended to increase and lactate decreased (*P*=0.089) from week 0 to week 7.

### Correlation of body fat accumulation and metabolic profiling data

The effects of the two diets on body fat distribution and body composition have been published previously.^6^ For the 35 individuals, the effects can be summarized as follows: Similar weight gain was measured in both groups during overfeeding, but PUFA caused a lower deposition of liver fat (*P*=0.077), visceral fat (*P*=0.073) and total body fat ( *P*=0.046) as compared with SFA. In contrast, lean tissue (*P*=0.044) was increased to a greater extent after overfeeding PUFA vs SFA. The effects of the two diets on body fat distribution and body composition are summarized in Table 1.

**Figure 4:**
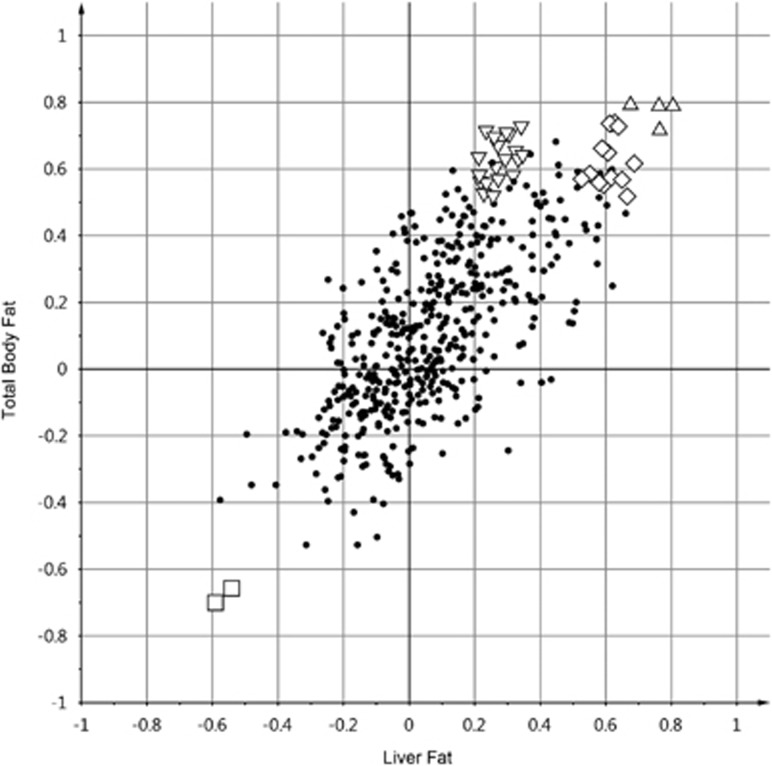
SUS plot for the comparison of the correlation of liver fat and total body fat with metabolic NMR data. Each symbol (including the dots) represents a spectral bin from the NMR data. This plot indicates a similar relationship for the metabolites 3-methyl-2-oxovalerate, (▴, triangles), 3-hydroxybutyrate (♦, diamonds) and alanine (▪, squares), while the BCAAs (▾, inverted triangles) have a higher correlation with the total body fat than with liver fat.

In the current study, four OPLS models were generated to study how each of these body compartments correlated with the NMR data, the models were evaluated using SUS plots. A SUS-plot based on the OPLS models of total body fat and liver fat is shown in Figure 4. The increase in total body fat and liver fat seemed to correlate with higher plasma levels of BCAAs, 3-hydroxybutyrate and 3-methyl-2-oxovalerate, and decreased levels of alanine. However, it could be argued that the BCAAs are unique structures for body fat because a much weaker correlation was observed for liver fat (Figure 4). Decreased lactate levels were also correlated with increased body fat.

Owing to the low predictability of the OPLS models (Figure 4), each metabolite was subjected to linear regression analysis. Metabolites having a *P*-value lower than 0.1 are presented in Table 2. The fat depots (liver fat, total body fat and visceral adipose tissue) showed similar relationships to lactate and alanine while there were some differences in correlations for the BCAAs, acetate and ketone bodies. Lean tissue exhibited a reverse relationship to valine, leucine and lactate compared with the fat depots.

## Discussion

A key finding in the LIPOGAIN trial was suppressed liver and visceral fat accumulation during weight gain by PUFA in comparison with that with SFA. By using an NMR-based metabolic profiling approach, we were able to identify a few non-lipid metabolites that differentiated between the diets; whether these findings might provide insights in the potential mechanisms behind the fatty-acid-specific effects on body fat deposition, however, requires further investigation. Differences in the plasma profiles between PUFA and SFA overfeeding were evident for some amino acids, ketone bodies and lactate, but overall, the differences were small and mostly not statistically significant. PUFA was associated with higher levels of lactate and lower levels of acetate and 3-hydroxybutyrate compared with SFA. Within the SFA group, a trend towards elevated levels of BCAAs and AAAs was observed. The fat depots (liver fat, total body fat and visceral adipose tissue) showed inverse relationships with lactate and alanine while there were some differences among fat depots concerning the positive correlations (BCAAs, acetate and ketone bodies). Lean tissue showed a direct relationship to lactate and alanine and an inverse reverse relationship to valine and leucine.

For the whole population, 3-hydroxybutyrate decreased, which confirms our previous results obtained by a kinetic enzymatic method.^6^ As this was a hypercaloric intervention, ketones would be expected to decrease. Interestingly, though, 3-hydroxybutyrate decreased numerically twice as much in the PUFA group (although only with 90% confidence). This was somewhat surprising, because, in general, PUFAs are known to be oxidized more easily than SFAs.^19^ However, this finding does not necessarily give an accurate reflection of the differential oxidation of individual fatty acids, which is often measured in acute feeding studies. Whether the different effect of PUFA and SFA on body composition might translate into differences in insulin sensitivity is not yet clear because we have not measured insulin sensitivity directly, and this therefore needs further investigation. In our previous report, surrogate measures of unspecific insulin sensitivity (homeostasis model assessment-estimated insulin resistance) did not differ significantly between diets.^6^ Nevertheless, there are several signs from the current metabolic profiling data which indicate that the insulin sensitivity of adipose tissue and liver did actually increase in the PUFA group in comparison with the SFA group. First, higher insulin sensitivity in the liver in the PUFA group might explain the stronger suppression of ketogenesis. Second, the significant decrease in acetate in the PUFA group might be attributable to the lower levels of non-essential fatty acids,^7^ implying a higher insulin sensitivity in adipose tissue, allowing for a stronger suppression of lipolysis. Third, the significant increase in lactate in the PUFA group might imply a higher reliance on carbohydrate oxidation (metabolic flexibility), potentially supporting a higher insulin sensitivity. Such an effect is, however, not clearly supported by crude measures of fasting glucose metabolism as previously reported, and thus needs confirmation.^6^ Fourth, some genes regulated by insulin (for example, IGF1) showed increased expression in adipose tissue in the PUFA group.^6^ Fifth, a trend towards elevated levels of branched and AAAs in the SFA group may be relevant, because elevated levels have been observed in obese and diabetic individuals compared with lean and non-diabetic individuals.^8, 9, 10, 20, 21^ Insulin is a major regulator of circulating BCAAs and dose-dependently lowers plasma BCAAs by stimulating their degradation in the liver.^22^ The mechanism is dependent on insulin signaling in the brain (hypothalamus), and short-term overfeeding was shown to impair the ability of insulin to lower plasma BCAAs.^22^ Elevated plasma BCAA levels have therefore been suggested to represent a marker of hypothalamic insulin resistance. Possibly, the increased plasma levels of BCAAs in the SFA group in the present study may reflect hypothalamic insulin resistance, a speculation that does require further studies. However, in the current study, changes in none of these metabolites were significantly associated with changes in homeostasis model assessment-estimated insulin resistance, implying that any potential effects on insulin resistance is subtle and may not be captured by unspecific measures such as homeostasis model assessment-estimated insulin resistance. Importantly, the achieved weight gain in the current study was small (1.6 kg) and the population remained lean after overfeeding and hence no major deteriorations in insulin sensitivity could be expected. Although not statistically significant, the apparent higher lactate levels during PUFA vs SFA treatment is of interest because recent data suggest that lactate could mediate a thermogenic effect through enhanced mitochondrial activity and UCP-1 expression in adipose tissue.^23^ Such a potential mechanism of a PUFA-induced mitochondrial fat combustion also accord with the inverse association between lactate and accumulation of visceral adipose tissue and total body fat in both groups combined. A potential role of PUFA-induced increase of lactate levels in reducing fat accumulation during overeating, however, requires further investigation.

Moreover, a correlation between circulating BCAAs and the fat depots was expected because higher BCAA levels has been observed in obese and diabetic individuals compared with lean and non-diabetic individuals.^8, 9, 10, 20, 21^ However, in the present study, we also found that the BCAAs leucine and valine were directly associated with the accumulation of visceral adipose tissue, and inversely associated with the accumulation of lean tissue during overfeeding. Also, a transformation product of isoleucine, 3-methyl-2-oxovalerate, showed weak positive associations with liver fat and total body fat in the current study. This metabolite has previously been associated with type 2 diabetes^24^ and insulin resistance.^25^

As the muffins contained two different types of fat, it was likely that a direct metabolic profiling study, including lipids, would miss the dietary effects on non-lipid metabolites. Furthermore, lipids and proteins may cause phase and/or baseline distortion, as well as interfering with the quantification of many non-lipid metabolites such as BCAAs. A non-lipid approach using precipitation with acetonitrile reduced the amount of lipids in the sample, thereby increasing the selectivity and enabling the semi-quantification of metabolites otherwise overlapped with the abundant peaks from the lipids and proteins (Figure 1). Another more established way to achieve a similar result would have been to use the Carr-Purcell-Meiboom-Gill pulse sequence.^26^ However, it is known that precipitation is a more efficient way to reduce the number of peaks from lipids and proteins.^27, 28, 29^ Another advantage with the preparation method presented is that it makes it possible to control the pH and ion strength, thus increasing the reproducibility.

Attaining adequate data quality is vital for achieving appropriate biological interpretations of metabolic data. In this study, each spectrum was visually inspected, where peak shape and abnormal peaks were of prior concern. Secondly, the PCA model was used to control the design of the study, the experimental setup, spectra quality and by looking for confounders (that is, gender, age, baseline body mass index, preparation date and acquisition date). As the model displayed no such patterns, the quality, taking into account both the design and the experimental conditions, was considered to be satisfactory. PCA and OPLS-DA models were used to identify metabolites that differed between the diets. In nutritional metabolomic studies, it is expected to have a very high inter-individual variability making it challenging to create reliable multivariate models. Therefore, as a validation of the models, each metabolite found as a contributor to the group classification was tested by using unpaired *t*-test. This approach therefore minimized the risk of interpreting over-fitted models.

In conclusion, by utilizing a non-lipid metabolic profiling approach, we enabled the identification and semi-quantification of important non-lipid metabolites and improved the quality of the spectra. Differences or trends in the plasma profiles between SFA and PUFA overfeeding were evident for amino acids, ketone bodies and lactate. Ketones and BCAA were associated with increased visceral, liver and total fat mass, whereas alanine and lactate showed an inverse relationship. Conversely, lean tissue correlated with higher levels of lactate but lower BCAA levels. Further investigations are warranted to clarify how these metabolite changes might contribute to changes in body composition and fat accumulation, and possibly also sub-clinical insulin resistance during overfeeding.

## Figures and Tables

**Table 1:** Liver fat and body composition before and after 7 weeks of PUFA or SFA overeating

	*PUFA (*n=*15) baseline*	*Mean absolute change*	*SFA (*n=*20) baseline*	*Mean absolute change*	P *value (*t*-test)*
Body weight, kg	67.4±2.2	1.2±0.3	64.9±1.59	1.5±0.3	0.560
BMI, kg m^−2^	21.7±0.6	0.40±0.09	19.9±0.3	0.45±0.07	0.702
Liver fat, % (MRI)	0.91 ±0.1	0.03±0.08	1.17±0.2	0.5±0.08	0.077
Lean tissue, L (MRI)	42.5±2.4	0.80±0.2	42.0±1.5	0.32±0.2	0.044
VAT, L (MRI)	1.20±0.2	0.090±0.06	0.80±0.09	0.21±0.04	0.073
Total body fat, L (MRI)	17.5±1.8	0.72±1.2	13.6±1.1	1.43±0.85	0.046

Abbreviations: BMI, body mass index; MRI, magnetic resonance imaging; PUFA, polyunsaturated fatty acid; SFA, saturated fatty acid; VAT, visceral adipose tissue. The presented data are means±the standard error of the mean. *P*-values are generated from unpaired two sided *t*-tests. Thirty-five individuals were included compared with 37 in the original study.^6^ The PUFA group included 10 males and 5 females while the the SFA group included 14 males and 6 females.

**Table 2:** Metabolites that correlate with the accumulation of fat depots and lean tissue according to the linear regression models

*MRI assessment*	*Positive correlations*			*Negative correlation*		
	*Metabolite*	r^2^	P	*Metabolite*	r^2^	P
Lean tissue	Lactate	0.1	0.07	Leucine	0.16	0.02
				Valine	0.16	0.02
						
Visceral adipose tissue	Leucine	0.16	0.02	Alanine	0.12	0.04
	Valine	0.16	0.02	Lactate^a^	0.06	0.14
						
Liver fat	3-methyl-2-oxovalerate	0.09	0.09	Alanine	0.08	0.09
						
Total body fat	3-hydroxybutyrate^a^	0.04	0.25	Alanine	0.18	0.01
	3-methyl-2-oxovalerate	0.16	0.02	Lactate^a^	0.06	0.15
	Acetate	0.11	0.06			
	Leucine	0.14	0.03			

Abbreviations: MRI, magnetic resonance imaging. Inclusion criteria for correlating metabolites; higher *P*(corr) than 0.5 and a higher variable importance plot (VIP) value than 2 in the OPLS models, together with a *P*-value lower than 0.1.

a Included owing to the high importance when group discrimination was considered.
